# Additive Value of Preoperative Sarcopenia and Lymphopenia for Prognosis Prediction in Localized Pancreatic Ductal Adenocarcinoma

**DOI:** 10.3389/fonc.2021.683289

**Published:** 2021-05-27

**Authors:** Christelle d’Engremont, Julienne Grillot, Julie Raillat, Dewi Vernerey, Lucine Vuitton, Stéphane Koch, Célia Turco, Bruno Heyd, Guillaume Mouillet, Quentin Jacquinot, Christophe Borg, Angélique Vienot

**Affiliations:** ^1^ Department of Gastroenterology and Nutrition, University Hospital of Besançon, Besançon, France; ^2^ Methodology and Quality of Life in Oncology Unit, University Hospital of Besançon, Besançon, France; ^3^ Department of Digestive Surgery and Liver Transplantation, University Hospital of Besançon, Besançon, France; ^4^ Department of Medical Oncology, University Hospital of Besançon, Besançon, France; ^5^ INSERM, EFS BFC, UMR1098, RIGHT, Interactions Greffon-Hôte-Tumeur/Ingénierie Cellulaire et Génique, University of Bourgogne Franche-Comté, Besançon, France; ^6^ Clinical Investigational Center, CIC-1431, University Hospital of Besançon, Besançon, France

**Keywords:** lymphopenia, sarcopenia, pancreatic ductal adenocarcinoma, preoperative, prognostic factor

## Abstract

**Background:**

Surgical resection with adjuvant chemotherapy is the only treatment that can provide long term survival in localized pancreatic ductal adenocarcinoma (LPDAC). Notwithstanding, recurrence occurs in the vast majority of patients and a better stratification of preoperative therapies is required. This study aimed to investigate preoperative immunological and nutritional factors to predict relapse-free survival (RFS) in patients with LPDAC.

**Methods:**

Analyses were derived from all consecutive LPDAC patients treated with surgical resection at Besancon University Hospital, France, between January 2006 and December 2014 (n=146). Biological and nutritional parameters were recorded before and after surgery. The association of 24 baseline parameters with RFS was evaluated using univariate and multivariate Cox analyses. Based on the final model, a prognostic score was developed.

**Results:**

Lymphocyte count and body composition were available for 94 patients. In multivariate analysis, preoperative lymphopenia and sarcopenia (or a low muscle mass) were identified as independent prognostic factors for RFS. The score determined three groups with a median RFS of 5.6 months (95% confidence interval [CI] = 4.3 to 9.6 months) for high-risk group, corresponding to patients with lymphopenia; 11.5 months (95%CI = 9.8 to 13.9 months), and 21.2 months (95%CI = 9.9 to 55.3 months), for intermediate-(patient with sarcopenia without lymphopenia), and low-risk groups (no risk factor), respectively (p <0.001). Preoperative sarcopenia predicts the occurrence of postoperative lymphopenia in patients with a preoperative lymphocyte count above 1,000/mm^3^ (p = 0.0029).

**Conclusions:**

Preoperative lymphopenia and sarcopenia are pejorative prognostic factors in LPDAC and should be considered in the preoperative evaluation to stratify death risk in patients with LPDAC.

## Introduction

Pancreatic ductal adenocarcinoma (PDAC) is one of the most aggressive cancers with a 5-year overall survival rate of 7% (1). While significant advances have been made in improving the prognosis for breast, colorectal, and prostate cancer, PDAC is projected to become the second leading cause of cancer-related deaths in 2030 (2). In localized PDAC (LPDAC), surgical resection followed by adjuvant chemotherapy is the only treatment that can provide long term survival up to 50 months (3, 4). However, the relapse rates observed in these studies are still over 80% (3). The clinical outcomes of this population are also influenced by post-operative mortality (3-5% in expert centers) and by the frequent occurrence of postoperative morbidities (20-30% of the patients) limiting access to adjuvant chemotherapy (5, 6). Tumor size, lymph node ratio, tumor differentiation, margin of resection are validated prognostic factors; however, they are only available postoperatively and cannot be used to predict disease recurrence before surgery (7). Identification of biomarkers available before surgery and correlated with the risk of death is an unmet medical need. Such biomarkers will avoid unnecessary surgery and might contribute to better select patients eligible for neoadjuvant chemotherapies.

Recently, biological parameters, mostly related to inflammation and immunological status, have been assessed: elevated C-reactive protein (CRP), increased levels of cytokines, high leukocyte counts, and low lymphocyte counts are measurable prognostic factors that might predict the course of the disease (8, 9). A large body of evidence supports the potent role of pre-operative lymphopenia to discriminate PDAC patients’ risk of death, in comparison with traditional histological parameters (10). Sarcopenia is another important parameter associated with postoperative complications, chemotherapy toxicities, and poor survival in cancers (11). Almost 20 to 65% of patients with LPDAC had preoperative sarcopenia (12), and a correlation with postoperative complications and worse survival was reported in some studies (13–16). Nevertheless, the additive value of sarcopenia on the prognostic role of lymphopenia in PDAC has never been elucidated.

This study aimed to characterize preoperative prognostic factors for relapse-free survival (RFS) in patients with LPDAC to explore how sarcopenia modulate the prognostic influence of lymphopenia in these patients.

## Methods

### Patients

All consecutive patients with histologically proven LPDAC treated by surgical resection at Besancon University Hospital, France, between January 2006 and December 2014 were involved. Pancreatectomy and systematic lymphadenectomy were performed as a curative intent in all patients. A relapse of the disease was defined radiologically with RECIST v1.1 criteria (17). Patients could have received adjuvant chemotherapy. All therapeutic decisions were discussed and validated during digestive oncology-dedicated multidisciplinary meetings. Follow-up of patients was performed every three months with clinical examination, blood analysis (including carbohydrate antigen 19-9 [CA19-9] and carcinoembryonic antigen [CEA]), and computed tomography (CT) scan. The study is in accordance with standard procedures in France with approval from the relevant institutional review boards. The database was registered and declared to the National French Commission for bioinformatics data and patient liberty (CNIL; No. of CNIL declaration: 1906173 v 0). A general informed consent was signed by all patients at the time of their first visit to the university hospital. This consent allows the use of their clinical, radiological, and biological data in the cohort study. The database was locked on November 3, 2017.

Demographics, cancer history, clinical, pathological, radiological parameters, as well as treatment outcomes, were retrospectively collected from medical records. Preoperative and postoperative (one month after surgery) biological (CRP, albumin, lymphocytes, neutrophils, CA19-9, CEA) and nutritional parameters were recorded, including body composition parameters (skeletal muscle) by CT scan. According to our previous research, lymphopenia was defined as a lymphocyte count below 1,000/mm^3^ (10). An underweight was defined by body mass index <18.5 kg/m² or <21 kg/m² over 70 years. For the assessment of skeletal muscle area, CT Digital Imaging and Communication in Medicine (DICOM) images at the third lumbar (L3) level were analyzed using NIH Image J1.47 to determine the indexed muscle area (IMA) excluding L3, by a single operator, blinded to patient information. Muscle area was normalized by height in squared meters (m²) and reported as the IMA (cm²/m²). The thresholds for defining sarcopenia (or low skeletal muscle mass) were 38.5 cm²/m² for women and 52.4 cm²/m² for men, according to Prado et al. (18).

### Statistical Analysis

Median value (interquartile range [IQR]) and frequency (percentage) were provided for the description of continuous and categorical variables, respectively. Medians and proportions were compared using Student’s t-test and Chi-square test (or Fisher’s exact test, as appropriate), respectively.

RFS was calculated from the date of surgery to the date of postoperative tumor relapse or death from any cause, or the date of the last follow-up, at which point data were censored. Overall survival (OS) was calculated from the date of surgery to the date of death from any cause. Survival data were censored at the last follow-up. OS and RFS were estimated using the Kaplan-Meier method and described using median or rate at specific time points with 95% confidence intervals (95%CI). Follow-up duration was calculated using a reverse Kaplan-Meier estimation when feasible (19).

Cox-proportional-hazard models were performed to estimate the hazard ratio (HR) and 95%CI for factors associated with RFS. The association of 24 baseline parameters with RFS was first assessed using univariate Cox analyses and then parameters with p <0.05 were entered into a final multivariate Cox regression model, after considering collinearity among variables with a correlation matrix. When used in continuous in the Cox model, a potential non-linear relationship between predictors and RFS was first investigated using the fractional polynomials method to determine the best transformation for continuous variables (20–22) and validated by the restricted cubic splines method with graphical evaluation. The assumption of proportionality was checked by plotting log-minus-log survival curves and by the cumulative martingale process plots.

The accuracy of the final model was verified regarding two parameters: discrimination and calibration. The predictive value and the discrimination ability of the final model were assessed with the Harrell’s concordance index (C-index) (22). Random samples of the population were used to derive 95%CI bootstrap percentile for the C-statistic. Calibration was assessed by visual examination of calibration plot at 6, 12, 24, and 48 months. Internal validation of the final model was performed with a bootstrap sample procedure.

The final model was used to establish a prognostic score allowing the preoperative estimation of RFS. To give a reasonable spread of risk, we chose to distinguish two levels of sarcopenia using IMA, according to their risk score level, which were identified based on cut points determined following two methods: the median value and the Cox’s method (23). Patient characteristics were compared between prognostic risk groups using Fisher-exact test and Kruskal-Wallis test for categorical and quantitative parameters, respectively. The prognostic score developed to estimate preoperative RFS was applied in the same population to evaluate preoperative OS, and RFS with postoperative parameters.

All analyses were performed using SAS version 9.4 (SAS Institute) and R software version 2.15.2 (R Development Core Team; http://www.r-project.org). Values of p <0.05 were considered statistically significant and all tests were two-sided. Details on the interpretation of important statistical concepts are given in the **Supplementary Methods**.

## Results

### Population Characteristics

**Figure 1:**
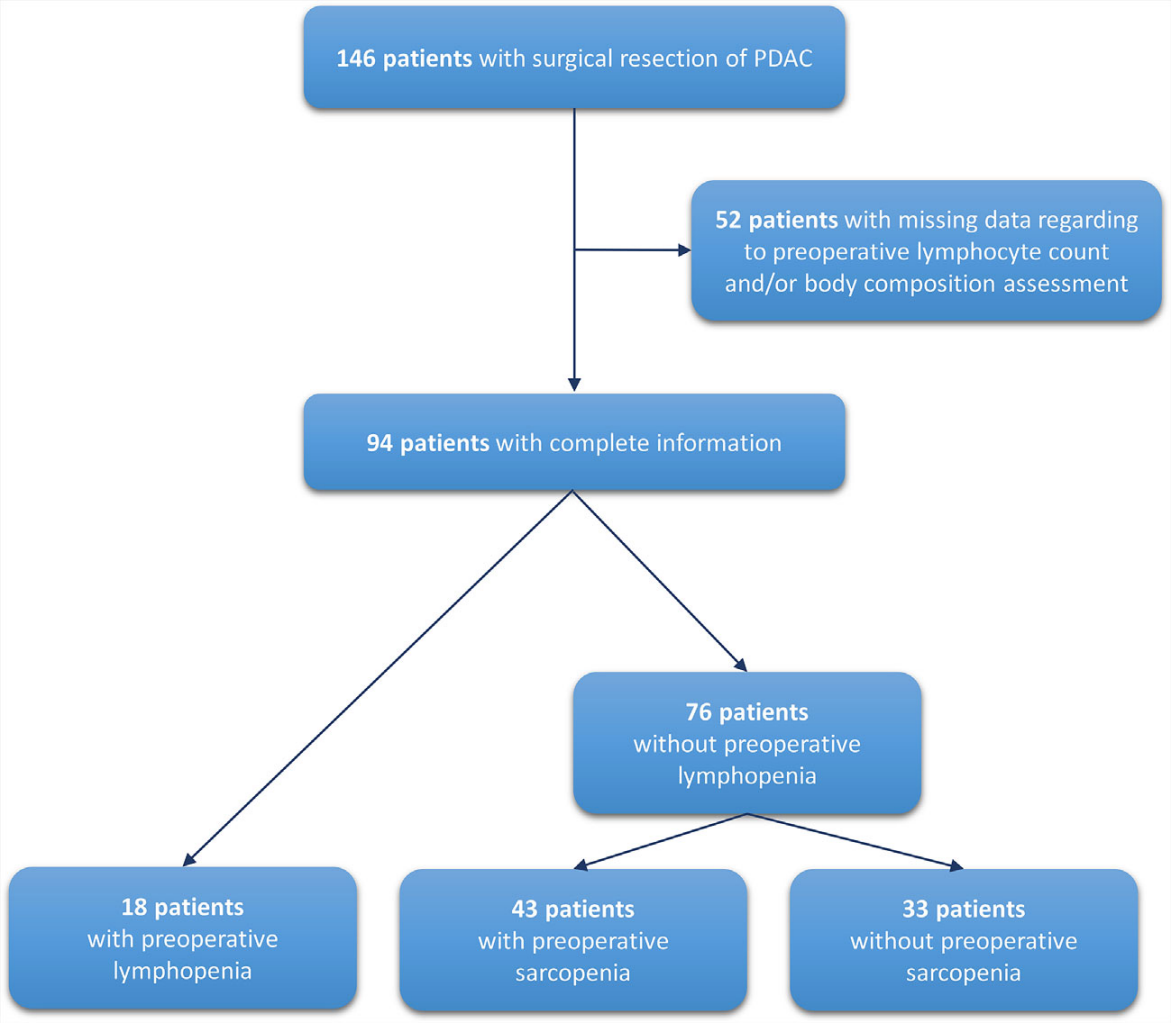
Flow chart.

From January 2006 to December 2014, 146 patients who underwent surgery for a LPDAC were included in this cohort (**Figure 1**). Patient characteristics are described in **Table 1**. The median age was 67.7 years (IQR, 61.8 – 73.8 years), 79 patients (54.1%) were men, 66 (49.2%) were never smokers, and 29 (20.0%) had diabetes history. LPDAC was localized in the head of the pancreas in 120 patients (82.2%). Jaundice was found in 89 patients (61.4%), among them 42 patients (29.0%) required biliary drainage before surgery. At the time of diagnosis, eight patients (5.5%) were underweight and 20 patients (18.7%) had hypoalbuminemia (<30 g/L), while the prevalence of reduced muscle mass was 59.2% (n=58), respectively. The median CRP/albumin ratio was 0.19 (IQR, 0.11 – 0.74). A lymphopenia was reported for 26 patients (18.7%), with a median neutrophil-to-lymphocyte ratio (NLR) at 2.86 (IQR, 2.14 – 4.40).

**Table 1:** Patient characteristics with surgical resection of PDAC.

Characteristics		Overall population (N = 146)	Patients with incomplete information (N = 52)	Patients with complete information (N = 94)	P^†^
**Demographics**					
**Age**, median [IQR], years		67.7 [61.8 – 73.8]	68.5 [62.5 – 75.1]	67.2 [60.7 – 72.7]	0.2519
**Sex**, No. (%)					0.5182
	Male	79 (54.1)	30 (57.7)	49 (52.1)	
** **	Female	67 (45.9)	22 (42.3)	45 (47.9)	
**Smoking status**, No. (%)					0.6840
** **	Never smoker	66 (49.2)	23 (46.9)	43 (50.6)	
** **	Former or current smoker	68 (50.8)	26 (53.1)	42 (49.4)	
** **	Missing	12	3	9	
**Alcohol consumption**, No. (%)					0.7697
** **	No	123 (90.4)	45 (91.8)	78 (89.7)	
** **	Yes	13 (9.6)	4 (8.2)	9 (10.3)	
** **	Missing	10	3	7	
**Diabetes**, No. (%)					0.4885
** **	No	116 (80.0)	40 (76.9)	76 (81.7)	
** **	Yes	29 (20.0)	12 (23.1)	17 (18.3)	
** **	Missing	1	0	1	
**Personal history of cancer**, No. (%)					0.9063
** **	No	122 (84.1)	44 (84.6)	78 (83.9)	
** **	Yes	23 (15.9)	8 (15.4)	15 (16.1)	
** **	Missing	1	0	1	
**Family history of cancer**, No. (%)					0.5120
** **	No	75 (57.3)	24 (53.3)	51 (59.3)	
** **	Yes	56 (42.8)	21 (46.7)	35 (40.7)	
** **	Missing	15	7	8	
**Preoperative parameters**					
**Tumor site**, No (%)					0.9064
	Head	120 (82.2)	43 (82.7)	77 (81.9)	
	Body and/or Tail	26 (17.8)	9 (17.3)	17 (18.1)	
**Tumor size**, median [IQR], cm		3.5 [3.0 – 4.5]	3.5 [3.0 – 4.5]	3.5 [3.0 – 4.5]	0.9243
	Missing	12	3	9	
**Jaundice**, No. (%)					0.5445
** **	No	56 (38.6)	18 (35.3)	38 (40.4)	
** **	Yes	89 (61.4)	33 (64.7)	56 (59.6)	
** **	Missing	1	1	0	
**Biliary drainage**, No (%)					0.9305
** **	No	103 (71.0)	36 (70.6)	67 (71.3)	
** **	Yes	42 (29.0)	15 (29.4)	27 (28.7)	
** **	Missing	1	1	0	
**Preoperative nutritional assessment**					
**Body mass index**, median [IQR], kg/m^2^		24.8 [22.2 – 27.7]	24.2 [21.9 – 26.9]	25.3 [22.7 – 28.0]	0.3172
**Body mass index**, No. (%), kg/m^2^					0.4000
** **	Normal weight (18.5–25)	68 (47.9)	28 (56.0)	40 (43.5)	
** **	Underweight (<18.5)	4 (2.8)	1 (2.0)	3 (3.2)	
** **	Overweight (25–30) and obesity (30)	70 (49.3)	21 (42.0)	49 (53.3)	
** **	Missing	4	2	2	
**Weight loss**, median [IQR], %		7.6 [4.0 – 12.0]	8.6 [4.5 – 12.9]	7.0 [3.8 – 11.8]	0.2494
	Missing	8	4	4	
**Indexed muscle area (IMA)**, median [IQR], cm²/m²		44.9 [37.9 – 51.3]	47.8 [45.1 – 55.4]	44.4 [37.9 – 51.3]	0.1992
	Missing	48	48	0	
**Sarcopenia**, No. (%)					0.6431
** **	No	40 (40.8)	1 (25.0)	39 (41.5)	
** **	Yes	58 (59.2)	3 (75.0)	55 (58.5)	
** **	Missing	48	48	0	
**Preoperative biological parameters**					
**Lymphocytes**, median [IQR], mm^3^		1500.0 [1034.0 – 1900.0]	1520.0 [1098.0 – 1900.0]	1490.0 [1034.0 – 1900.0]	0.7344
	Missing	7	7	0	
**Lymphopenia (<1000/mm^3^)**, No. (%)					0.8462
** **	No	113 (81.3)	37 (82.2)	76 (80.9)	
** **	Yes	26 (18.7)	8 (17.8)	18 (19.1)	
** **	Missing	7	7	0	
**Neutrophil-to-lymphocyte ratio**, median [IQR]		2.86 [2.14 – 4.40]	3.3 [2.4 – 5.0]	2.7 [2.0 – 3.7]	0.0888
	Missing	11	9	2	
**CA19-9**, median [IQR], UI/mL		127.5 [40.0 – 485.0]	237.0 [59.0 – 800.0]	110.0 [38.0 – 354.5]	0.0464
	Missing	28	14	14	
**CEA**, median [IQR], ng/mL		2.0 [2.0 – 4.6]	2.0 [2.0 – 4.4]	2.0 [1.9 – 5.0]	0.9218
	Missing	53	25	28	
**C-reactive protein**, median [IQR], mg/L		6.5 [3.5 – 19.9]	6.0 [3.0 – 22.0]	7.0 [3.8 – 19.9]	0.6204
	Missing	37	17	20	
**C-reactive protein**, No. (%), mg/L					0.9165
** **	<5	35 (32.1)	11 (31.4)	24 (32.4)	
** **	≥5	74 (67.9)	24 (68.6)	50 (67.6)	
** **	Missing	37	17	20	
**Albumin**, median [IQR], g/L		35.0 [30.8 – 38.9]	34.0 [30.0 – 38.0]	35.0 [31.0 – 39.0]	0.5664
	Missing	39	17	22	
**Albumin**, No. (%), g/L					0.8087
** **	<30	20 (18.7)	7 (20.0)	13 (18.1)	
** **	≥30	87 (81.3)	28 (80.0)	59 (81.9)	
** **	Missing	39	17	22	
**C-reactive protein/albumin ratio**, median [IQR]		0.19 [0.11 – 0.74]	0.19 [0.09 – 0.65]	0.19 [0.12 – 0.77]	0.5718
	Missing	54	23	31	
**Surgical parameters**					
**Time between diagnosis and surgery**, median [IQR], days		25.5 [14.0 – 42.0]	34.5 [18.0 – 50.0]	23.0 [11.0 – 40.0]	0.0403
	Missing	18	18	0	
**Length of stay**, median [IQR], days		21.0 [16.0 – 30.0]	20.0 [15.0 – 32.0]	21.0 [17.0 – 30.0]	0.3876
	Missing	1	1	0	
**Complications**, No. (%)					0.3801
** **	No	103 (70.5)	39 (75.0)	64 (68.1)	
** **	Yes	43 (29.5)	13 (25.0)	30 (31.9)	
**Pathologic parameters**					
**pT local invasion**, No. (%)					0.0841
	0 – 1	5 (3.6)	4 (8.2))	1 (1.1)	
	2	15 (10.7)	5 (10.2)	10 (11.0)	
	3	111 (79.3)	37 (75.5)	74 (81.3)	
	4	9 (6.4)	3 (6.1)	6 (6.6)	
** **	Missing	6	3	3	
**pN status**, No. (%)					0.8377
** **	0	21 (14.6)	8 (15.4)	13 (14.1)	
** **	1	123 (85.4)	44 (84.6)	79 (85.9)	
** **	Missing	2	0	2	
**Number of positive lymph nodes**, median [IQR]		2.0 [1.0 – 5.0]	2.0 [1.0 – 5.0]	3.0 [1.0 – 5.0]	0.7485
	Missing	2	0	2	
**Number of lymph nodes removed**, median [IQR]		16.0 [11.0 – 21.5]	13.5 [9.5 – 17.5]	18.0 [12.0 – 23.0]	0.0217
	Missing	2	0	2	
**Lymph node ratio**, median [IQR]		0.17 [0.07 – 0.31]	0.18 [0.08 – 0.41]	0.16 [0.05 – 0.27]	0.1944
	Missing	3	0	3	
**Extracapsular invasion**, No. (%)					0.5821
** **	No	91 (66.9)	32 (64.0)	59 (68.6)	
** **	Yes	45 (33.1)	18 (36.0)	27 (31.4)	
** **	Missing	10	2	8	
**Vascular invasion**, No. (%)					0.9450
** **	No	57 (40.4)	20 (40.8)	37 (40.2)	
** **	Yes	84 (59.6)	29 (59.2)	55 (59.8)	
** **	Missing	5	3	2	
**Lymphatic invasion**, No. (%)					0.6157
** **	No	76 (52.8)	26 (50.0)	50 (54.4)	
** **	Yes	68 (47.2)	26 (50.0)	42 (45.6)	
** **	Missing	2	0	2	
**Residual tumor**, No. (%)					0.4849
** **	0	114 (79.2)	41 (78.9)	73 (79.4)	
** **	1	29 (20.1)	10 (19.2)	19 (20.6)	
** **	2	1 (0.7)	1 (1.9)	0 (0.0)	
** **	Missing	2	0	2	
**Histological grade**, No. (%)					0.5611
** **	Poorly differentiated or undifferentiated	18 (17.0)	7 (20.0)	11 (15.5)	
** **	Well or moderately differentiated	88 (83.0)	28 (80.0)	60 (84.5)	
** **	Missing	40	17	23	
**Postoperative nutritional assessment**					
**Body mass index**, median [IQR], kg/m^2^		22.5 [20.1 – 25.0]	21.7 [16.6 – 24.7]	22.8 [20.8 – 25.2]	0.2177
**Body mass index**, No. (%), kg/m^2^					0.7069
** **	Normal weight (18.5–25)	88 (63.8)	31 (64.6)	57 (63.3)	
** **	Underweight (<18.5)	14 (10.1)	6 (12.5)	8 (8.9)	
** **	Overweight (25–30) and obesity (30)	36 (26.1)	11 (22.9)	25 (27.8)	
** **	Missing	8	4	4	
**Weight loss**, median [IQR], %		15.5 [11.3 – 22.0]	17.7 [13.0 – 22.2]	15.0 [9.6 – 20.6]	0.1553
	Missing	12	6	6	
**Indexed muscle area (IMA)**, median [IQR], cm²/m²		43.6 [39.7 – 48.8]	44.1 [40.3 – 48.1]	43.4 [39.4 – 48.9]	0.4069
	Missing	56	27	29	
**Sarcopenia**, No. (%)					0.5688
** **	No	33 (36.7)	8 (32.0)	25 (38.5)	
** **	Yes	57 (63.3)	17 (68.0)	40 (61.5)	
** **	Missing	56	27	29	
**Postoperative biological parameters**					
**Lymphocytes**, median [IQR], mm^3^		1500.0 [1010.0 – 1952.0]	1400.0 [955.0 – 1981.0]	1561.5 [1069.0 – 1950.0]	0.4382
	Missing	16	8	8	
**Lymphopenia (<1000/mm^3^)**, No. (%)					0.3307
** **	No	101 (77.7)	32 (72.7)	69 (80.2)	
** **	Yes	29 (22.3)	12 (27.3)	17 (19.8)	
** **	Missing	16	8	8	
**Neutrophil-to-lymphocyte ratio**, median [IQR]		3.11 [1.77 – 5.27]	3.5 [2.4 – 5.6]	2.87 [1.67 – 4.85]	0.1058
	Missing	28	10	18	
**CA19-9**, median [IQR], UI/mL		21.9 [7.0 – 71.4	37.0 [11.3 – 258.0]	19.0 [6.2 – 55.1]	0.0571
	Missing	59	21	38	
**C-reactive protein**, median [IQR], mg/L		15.2 [7.6 – 52.5]	11.9 [6.0 – 58.0]	17.0 [8.0 – 52.0]	0.4250
	Missing	46	22	24	
**C-reactive protein**, No. (%), mg/L					1.0000
** **	<5	5 (9.3)	2 (11.1)	3 (8.3)	
** **	≥5	49 (90.7)	16 (88.9)	33 (91.7)	
** **	Missing	46	22	24	
**Albumin**, median [IQR], g/L		29.0 [24.0 – 34.0]	32.0 [25.0 – 35.0]	28.0 [24.0 – 34.0]	0.2034
	Missing	59	27	32	
**Albumin**, No. (%), g/L					0.2924
** **	<30	46 (52.9)	11 (44.0)	35 (56.5)	
** **	≥30	41 (47.1)	14 (56.0)	27 (43.5)	
** **	Missing	59	27	32	
**C-reactive protein/albumin ratio**, median [IQR]		0.67 [0.25 – 2.10]	0.56 [0.21 – 2.01]	0.80 [0.30 – 2.21]	0.5348
	Missing	67	30	37	
**Follow-up parameters**					
**Median follow-up time** [IQR], months		89.5 [77.5 – 99.4]	All patients were followed until death (maximum time observed = 131.8 months) except 17 censored patients with a median follow-up equal to 17.1 months	45.3 [35.0 – 86.7]	

^†^χ2 tests or Fisher’s exact tests used to compare proportions, and Wilcoxon tests used to compare continuous variables between the groups with or without complete information regarding to lymphopenia and sarcopenia.

All statistical tests were two-sided.

IQR, Interquartile Range; CA 19-9, Carbohydrate Antigen 19-9; CEA, carcinoembryonic antigen; NA, not available.

The median time between diagnosis and surgery was 25.5 days (IQR, 14.0 – 42.0 days), and the median hospitalization duration for surgery was 21.0 days (IQR, 16.0 – 30.0 days). The median tumor size was 3.5 cm (IQR, 3.0 – 4.5 cm) and the complete surgical removal rate (R0) was 79.2%. In the postoperative setting, 57 patients (63.3%) had low muscle mass and 46 patients (52.9%) had hypoalbuminemia. The prevalence of lymphopenia was 22.3% (n=29) after surgery. Adjuvant chemotherapy was administered for 114 patients (78.1%) within 62.5 days (IQR, 51.0 – 77.0 days) after surgery, mostly with gemcitabine (n=107; 93.9%). After a median duration of 5.1 months (IQR, 2.8 – 5.3 months), adjuvant chemotherapy was discontinued for 46 patients (40.3%), mainly due to toxicities (42.1%) or progression disease (34.2%). Cancer relapse was metastatic in 78 patients (85.7%). The median time of follow-up was 89.5 months (IQR, 77.5 – 99.4 months).

### Preoperative Prognostic Factors of RFS

We identified six preoperative parameters as prognostic factors for RFS, in the univariate analyses (p <0.05): tumor size, IMA, sarcopenia, lymphopenia, NLR, and CA19-9 (**Table 2**). Other nutritional factors (weight loss or albumin level) were not statistically associated with RFS. The transformations used for continuous variables are summarized in **Supplementary Figure 1**. A square root transformation was applied for NLR and CA 19-9, while an inverse square root transformation was necessary for lymphocyte count. All other continuous variables were considered without any transformation.

**Table 2:** Prognostic factors associated with relapse-free survival in univariate analysis.

Parameters		No. of patients	No. of events	HR (95% CI)	P^†^
**Demographic parameters**					
**Age**, years		146	99	0.993 (0.973 – 1.014)	0.5032
**Sex**					
	Male	79	52	1.00 (Reference)	
** **	Female	67	47	1.303 (0.876 – 1.938)	0.1922
**Smoking status**					
** **	Never smoker	66	45	1.00 (Reference)	
** **	Former or current smoker	68	49	0.952 (0.635 – 1.429	0.8134
** **	Missing	12	5		
**Alcohol consumption**					
** **	No	123	88	1.00 (Reference)	
** **	Yes	13	8	0.825 (0.400 – 1.704)	0.6039
** **	Missing	10	3		
**Diabetes**					
** **	No	116	77	1.00 (Reference)	
** **	Yes	29	22	1.302 (0.808 – 2.099)	0.2781
** **	Missing	1	0		
**Personal history of cancer**					
** **	No	122	86	1.00 (Reference)	
** **	Yes	23	13	0.795 (0.444 – 1.426)	0.4422
** **	Missing	1	0		
**Family history of cancer**					
** **	No	75	52	1.00 (Reference)	
** **	Yes	56	40	0.733 (0.484 – 1.110)	0.1426
** **	Missing	15	7		
**Family history of pancreatic cancer**					
** **	No	124	87	1.00 (Reference)	
** **	Yes	7	5	1.177 (0.477 – 2.903)	0.7241
** **	Missing	15	7		
**Preoperative parameters**					
**Tumor site**					
	Head	120	81	1.00 (Reference)	
	Body and/or Tail	26	18	1.183 (0.708 – 1.976)	0.5209
**Tumor size**, cm		134	94	1.186 (1.022 – 1.376)	0.0248
** **	Missing	12	5		
**Jaundice**					
** **	No	56	36	1.00 (Reference)	
** **	Yes	89	63	1.039 (0.689 – 1.565)	0.8565
** **	Missing	1	0		
**Biliary drainage**					
** **	No	103	66	1.00 (Reference)	
** **	Yes	42	33	1.520 (0.999 – 2.315)	0.0508
** **	Missing	1	0		
**Preoperative nutritional assessment**					
**Body mass index**, kg/m^2^					
** **	Normal weight (18.5–25)	68	44	1.00 (Reference)	
** **	Underweight (<18.5)	4	3	0.973 (0.301 – 3.144)	
** **	Overweight (25–30) and obesity (30)	70	49	0.874 (0.581 – 1.314)	0.8085
** **	Missing	4	3		
**Weight loss**, %		138	94	1.007 (0.975 – 1.039)	0.6813
	Missing	8	5		
**Indexed muscle area (IMA)**, median [IQR], kg/m^2^		98	67	0.963 (0.935 – 0.991)	0.0110
	Missing	48	32		
**Sarcopenia**					
** **	No	40	26	1.00 (Reference)	
** **	Yes	58	41	1.773 (1.075 – 2.923)	0.0248
** **	Missing	48	32		
**Preoperative biological parameters**					
**Neutrophils**, mm^3^		135	92	1.000 (1.000 – 1.000)	0.4355
	Missing	11	7		
**Lymphocytes**, mm^3^ (square root inverse transformation value)		139	94	3.193E17 (18.336 – 5.56E33)	0.0347
	Missing	5	5		
**Lymphopenia**					
** **	No	113	72	1.00 (Reference)	
** **	Yes	26	22	2.811 (1.710 – 4.620)	<0.0001
** **	Missing	5	5		
**Neutrophil-to-lymphocyte ratio** (square root value)		135	92	1.487 (1.006 – 2.200)	0.0468
	Missing	9	7		
**CA19-9**, UI/mL (square root value)		118	83	1.009 (1.001 – 1.018)	0.0372
	Missing	28	16		
**CEA**, ng/mL		93	65	1.006 (0.993 – 1.020)	0.3692
	Missing	53	34		
**C-reactive protein**, mg/L		109	76	1.003 (0.994 – 1.013)	0.5061
	Missing	37	23		
**C-reactive protein**, mg/L					
** **	<5	35	22	1.00 (Reference)	
** **	≥5	74	54	1.158 (0.705 – 1.903)	0.5616
** **	Missing	37	23		
**Albumin**, g/L		107	76	0.982 (0.940 – 1.025)	0.3953
	Missing	39	23		
**C-reactive protein/albumin ratio**		92	64	1.141 (0.853 – 1.526)	0.3751
	Missing	54	35		

^†^Cox proportional hazard models used to estimate association of the parameters with overall survival. Values of P <.05 were considered statistically significant, and all tests were two-sided.

IQR, Interquartile Range; CA 19-9, Carbohydrate Antigen 19-9; CEA, carcinoembryonic antigen; HR, hazard ratio; CI, confidence interval.

A correlation matrix was used to detect statistically significant correlations between investigated parameters (**Supplementary Figure 2**). Significant correlations were defined by a correlation coefficient ≥ 0.4 associated with a p-value <0.001. A correlation was identified between “Lymphopenia” and “Neutrophil-to-lymphocyte ratio”, and between “Sarcopenia” and “Index Muscle Area”. We selected as most clinically relevant variables “Lymphopenia” and “Sarcopenia”.

Finally, the multivariable Cox analysis showed two independent risk factors for RFS: sarcopenia (HR = 1.78, 95% CI= 1.01 to 3.14, p = 0.0469) and lymphopenia (HR = 4.57, 95% CI = 2.24 to 9.34, p <0.0001; **Table 3**). Among the 146 patients operated for LPDAC, lymphocyte count and body composition calculation were available for 94 patients (**Figure 1**). However, the two groups with or without complete information displayed similar RFS (**Supplementary Figure 3**), and patient characteristics were well-balanced between them, except for the median time between diagnosis and surgery (**Table 1**).

**Table 3:** Preoperative prognostic factors associated with relapse-free survival in multivariable analysis (N = 72)^†^.

Parameters	No. of patients	No. of events	HR (IC à 95%)	P^‡^	Internal validation BCA HR 95%
**Tumor size**, cm	72	53	1.039 (0.832 – 1.297)	0.7364	0.773 – 1.398
**Sarcopenia**					
No	32	21	1.00 (Reference)		
Yes	40	32	1.779 (1.008 – 3.139)	0.0469	0.986 – 3.390
**Lymphopenia**					
No	15	14	1.00 (Reference)		
Yes	57	39	4.573 (2.240 –9.336)	<0.0001	2.054 – 9.119
**CA19-9**, UI/mL (square root value)	72	53	1.012 (0.992 – 1.032)	0.2399	0.986 – 1.030

^†^The final multivariable Cox model was obtained by entering risks factors from the univariate model that achieved P = .05 as the thresholds in a single multivariable proportional hazards model.

HR, hazard ratio; CI, confidence interval; BCA, accelerated bootstrap confidence interval; CA 19-9, Carbohydrate Antigen 19-9.

^‡^Cox proportional hazard models used to estimate association of the parameters with overall survival. Values of P <.05 were considered statistically significant, and all tests were two-sided.

### Performance Assessment and Internal Validation of the Final Model

The multivariable model exhibited good discrimination ability (C-index = 0.67, 95% CI = 0.57 to 0.77). The calibration plots showed an optimal agreement between model prediction and actual observation for predicting RFS probability at 6, 12, 24, and 48 months (**Supplementary Figure 4**). In the internal validation, uncertainties around hazard ratio measured with a bootstrapping procedure reflected the robustness of the final model (**Table 3**).

### Preoperative Scoring System to Predict RFS

**Figure 2:**
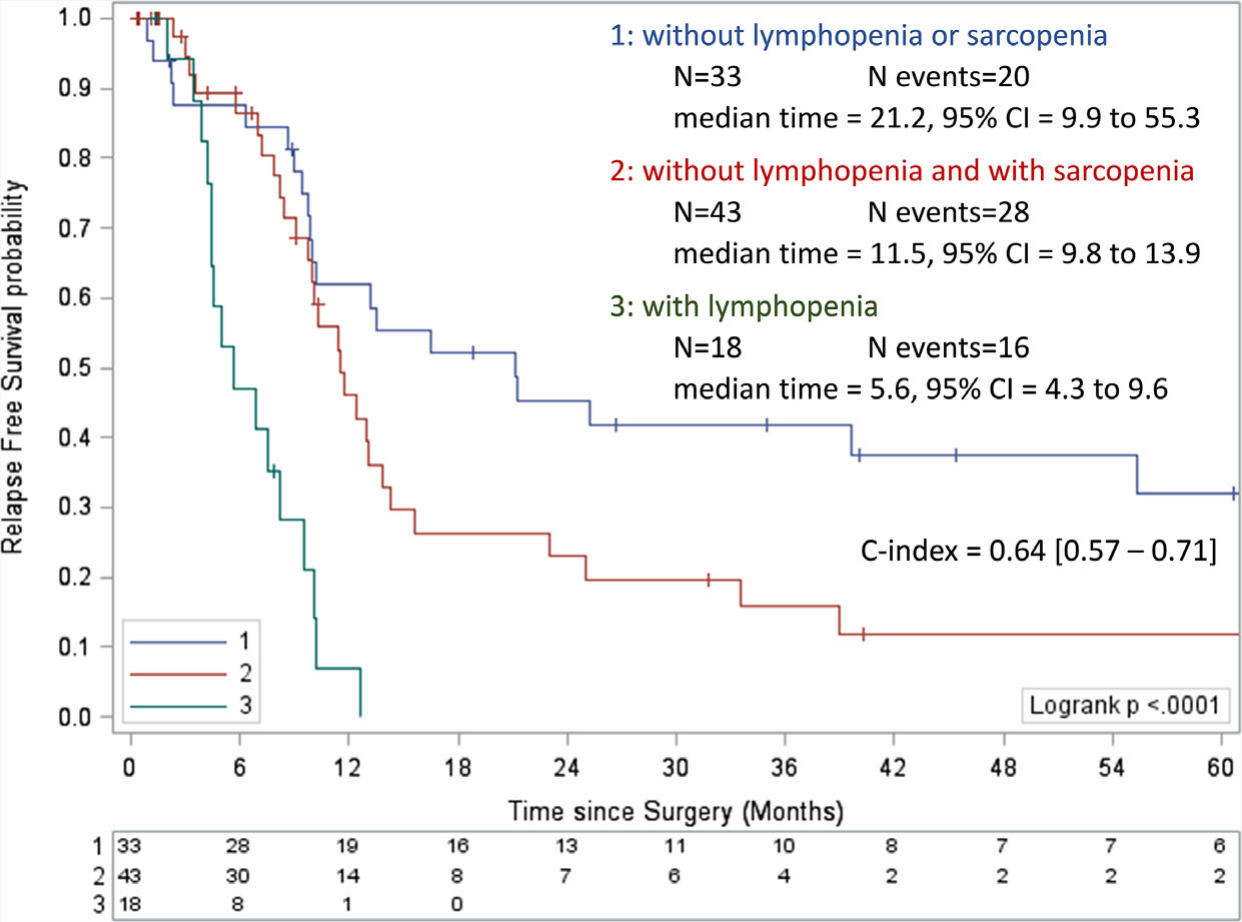
Kaplan-Meier curves of relapse-free survival according to preoperative lymphopenia and sarcopenia. Values of the log-rank test P <0.05 were considered statistically significant, and all tests were two-sided. CI, confidence interval.

A prognostic score integrating the two independent factors for RFS was built. Kaplan-Meier curves of RFS according to preoperative lymphopenia and sarcopenia showed four groups (**Supplementary Figure 5**). The survival of patients with lymphopenia (corresponding to the highest HR), with or without sarcopenia, was similar (median of 6.6 months, 95% CI = 4.4 to 12.6, and 5.6 months, 95%CI = 3.4 to 9.6, respectively). Thus, overall patients with lymphopenia were grouped together, and patients were categorized into three risk groups (high, intermediate, and low risk; **Figure 2**). The high-risk group is constituted by patients with lymphopenia and/or sarcopenia. Patients with sarcopenia without lymphopenia were classified in the intermediate group, while patients without any risk factor were in the low-risk group. Risk groups had median RFS of 5.6 months (95% CI = 4.3 to 9.6 months), 11.5 months (95% CI = 9.8 to 13.9 months), and 21.2 months (95% CI = 9.9 to 55.3 months), respectively (p <0.001).

Patient characteristics in each risk group are described in **Table 4**. The parameters were similar in the three groups, especially hypoalbuminemia. Patients with preoperative lymphopenia and/or sarcopenia had sarcopenia after surgery in 76.9%, 80.0%, and 27.3%, respectively in high, intermediate, and low risk groups.

**Table 4:** Patient characteristics in each risk group.

Characteristics		Patients with lymphopenia (N = 18)	Patients without lymphopenia		P^†^	Patients without lymphopenia		P^‡^
			With sarcopenia (N = 43)	Without sarcopenia (N = 33)		With high sarcopenia (N = 34)	With low sarcopenia (N = 9)	
**Demographics**								
**Age**, median [IQR], years		63.6 [58.5 – 69.8]	66.1 [661.9 – 71.0]	70.4 [61.8 – 74.5]	0.1043	64.9 [60.8 – 69.3]	68.1 [65.1 – 71.0]	0.3020
**Sex**, No. (%)					0.6352			0.0551
	Male	10 (55.6)	24 (55.8)	15 (45.4)		16 (47.1)	8 (88.9)	
** **	Female	8 (44.4)	19 (44.2)	18 (54.6)		18 (52.9)	1 (11.1)	
**Smoking status**, No. (%)					0.5081			0.4264
** **	Never smoker	11 (61.1)	16 (44.4)	16 (51.6)		14 (48.3)	2 (28.6)	
** **	Former or current smoker	7 (38.9)	20 (55.6)	15 (48.4)		15 (51.7)	5 (71.4)	
** **	Missing	0	7	2		5	2	
**Alcohol consumption**, No. (%)					0.0808			0.5916
** **	No	18 (100.0)	31 (81.6)	29 (93.6)		26 (83.9)	5 (71.4)	
** **	Yes	0 (0.0)	7 (18.4)	2 (6.4)		5 (16.1)	2 (28.6)	
** **	Missing	0	5	2		3	2	
**Diabetes**, No. (%)					0.0407			0.3193
** **	No	11 (61.1)	36 (85.7)	29 (87.9)		30 (88.2)	6 (75.0)	
** **	Yes	7 (38.9)	6 (14.3)	4 (12.1)		4 (11.8)	2 (25.0)	
** **	Missing	0	1	0		0	1	
**Preoperative parameters**								
**Tumor site**, No (%)					0.9839			0.1710
	Head	15 (83.3)	35 (81.4)	27 (81.8)		26 (76.5)	9 (100.0)	
	Body and/or Tail	3 (16.7)	8 (18.6)	6 (18.2)		8 (23.5)	0 (0.0)	
**Tumor size**, median [IQR], cm		3.5 [3.0 – 4.0]	3.5 [3.0 – 5.0]	3.5 [3.0 – 4.5]	0.4265	3.5 [2.7 – 5.0]	3.5 [3.0 – 4.5]	0.5731
	Missing	1	5	3		3	2	
**Jaundice**, No. (%)					0.1217			1.0000
** **	No	6 (33.3)	14 (32.6)	18 (54.6)		11 (32.3)	3 (33.3)	
** **	Yes	12 (66.7)	29 (67.4)	15 (45.4)		23 (67.7)	6 (66.7)	
**Preoperative nutritional assessment**								
**Body mass index**, No. (%), kg/m^2^					0.1764			0.3566
** **	Normal weight (18.5–25)	9 (50.0)	21 (50.0)	10 (31.2)		18 (54.6)	3 (33.3)	
** **	Underweight (<18.5)	1 (5.6)	2 (4.8)	0 (0.0)		2 (6.0)	0 (0.0)	
** **	Overweight (25–30) and obesity (30)	8 (44.4)	19 (45.2)	22 (68.8)		13 (39.4)	6 (66.7)	
** **	Missing	0	1	1		1	0	
**Weight loss**, median [IQR], %		6.5 [1.6 – 11.9]	7.8 [4.7 – 12.0]	6.1 [3.6 – 10.2]	0.5589	7.8 [4.7 – 12.0]	7.4 [4.7 – 10.3]	0.7425
	Missing	1	2	1		2	0	
**Indexed muscle area (IMA)**, median [IQR], cm²/m²		40.8 [37.9 – 48.5]	39.3 [36.4 – 46.6]	47.9 [43.0 – 57.6]	<0.0001	37.5 [35.3 – 43.9]	51.3 [49.3 – 51.9]	0.0002
**Preoperative biological parameters**								
**Neutrophil-to-lymphocyte ratio**, median [IQR]		5.38 [4.24 – 7.22]	2.51 [1.82 – 3.37]	2.72 [1.71 – 3.19]	<0.0001	2.54 [1.79 – 3.26]	2.51 [1.89 – 3.89]	0.5938
	Missing	1	0	1		0	0	
**CA19-9**, median [IQR], UI/mL		102.0 [37.0 – 364.0]	108.0 [39.0 – 398.0]	130.0 [18.0 – 345.0]	0.9941	94.5 [39.0 – 253.0]	120.0 [55.0 – 398.0]	0.6403
	Missing	3	8	3		4	4	
**CEA**, median [IQR], ng/mL		2.0 [1.2 – 3.0]	2.0 [1.8 – 4.1]	3.5 [2.0 – 5.0]	0.0216	2.0 [1.5 – 3.1]	3.3 [2.0 – 7.1]	0.2174
	Missing	5	15	8		10	5	
**C-reactive protein**, No. (%), mg/L					0.7973			0.5515
** **	<5	5 (38.5)	12 (33.3)	7 (28.0)		9 (31.0)	3 (42.9)	
** **	≥5	8 (61.5)	24 (66.7)	18 (72.2)		20 (69.0)	4 (57.1)	
** **	Missing	5	7	8		5	2	
**Albumin**, No. (%), g/L					0.9216			1.0000
** **	<30	3 (21.4)	6 (16.7)	4 (18.2)		5 (17.9)	1 (12.5)	
** **	≥30	11 (78.6)	30 (83.3)	18 (81.8)		23 (82.1)	7 (87.5)	
** **	Missing	4	7	11		6	1	
**C-reactive protein/albumin ratio**, median [IQR]		0.40 [0.12 – 1.05]	0.24 [0.10 -0.60]	0.19 [0.12 – 0.77]	0.8324	0.21 [0.13 – 0.44]	0.38 [0.02 – 0.94]	0.7518
	Missing	6	11	14		9	2	
**Surgical parameters**								
**Time between diagnosis and surgery**, median [IQR], days		29.0 [19.0 – 40.0]	24.0 [9.0 – 43.0]	19.0 [9.0 – 31.0]	0.3613	29.0 [9.0 – 48.0]	14.0 [9.0 – 24.0]	0.3752
**Length of stay**, median [IQR], days		24.0 [19.0 – 32.0]	22.0 [17.0 – 30.0]	19.0 [16.0 – 24.0]	0.1557	21.0 [17.0 – 29.0]	24.0 [19.0 – 36.0]	0.3293
**Complications**, No. (%)					0.0923			1.0000
** **	No	16 (88.9)	26 (60.5)	22 (66.7)		21 (61.8)	5 (55.6)	
** **	Yes	2 (11.1)	17 (39.5)	11 (33.3)		13 (38.2)	4 (44.4)	
**Postoperative nutritional assessment**								
**Body mass index**, No. (%), kg/m^2^					0.2678			0.2288
** **	Normal weight (18.5–25)	13 (72.2)	25 (64.1)	19 (57.6)		21 (70.0)	4 (44.4)	
** **	Underweight (<18.5)	2 (11.1)	5 (12.8)	1 (3.0)		4 (13.3)	1 (11.1)	
** **	Overweight (25–30) and obesity (30)	3 (16.7)	9 (23.1)	13 (39.4)		5 (16.7)	4 (44.4)	
** **	Missing	0	4	0		4	0	
**Weight loss**, median [IQR], %		14.3 [9.2 – 21.7]	15.4 [8.5 – 22.2]	14.8 [12.7 – 19.8]	0.9918	15.5 [9.2 – 22.2]	14.0 [6.2 – 17.6]	0.3565
	Missing	1	4	1		4	0	
**Indexed muscle area (IMA)**, median [IQR], cm²/m²		44.0 [40.7 – 49.0]	40.5 [37.6 – 43.8]	48.2 [43.4 – 51.3]	0.0018	38.9 [35.0 – 43.1]	46.2 [40.9 – 53.5]	0.0137
	Missing	5	13	11		11	2	
**Sarcopenia**, No. (%)					0.0003			0.1201
** **	No	3 (23.1)	6 (20.0)	16 (72.7)		3 (13.0)	3 (42.9)	
** **	Yes	10 (76.9)	24 (80.0)	6 (27.3)		20 (87.0)	4 (57.1)	
** **	Missing	5	13	11		11	2	
**Postoperative biological parameters**								
**Lymphopenia (<1000/mm^3^)**, No. (%)					0.0029			0.1563	
** **	No	9 (52.9)	30 (81.1)	30 (93.8)		25 (86.2)	5 (62.5)	
** **	Yes	8 (47.1)	7 (18.9)	2 (6.2)		4 (13.8)	3 (37.5)	
** **	Missing	1	6	1		5	1	
**Neutrophil-to-lymphocyte ratio**, median [IQR]		3.78 [2.06 – 5.13]	2.71 [1.58 – 4.20]	2.73 [1.59 – 5.27]	0.5571	2.64 [1.61 – 3.82]	5.50 [1.58 – 12.00]	0.2400
	Missing	3	8	7		6	2	
**CA19-9**, median [IQR], UI/mL		24.0 [17.0 – 74.0]	23.2 [7.0 – 67.9]	15.3 [5.4 – 29.0]	0.4298	26.2 [7.3 – 70.0]	8.0 [7.0 – 16.7]	0.1644
	Missing	9	17	12		13	4	
**C-reactive protein**, No. (%), mg/L					1.0000			1.0000
** **	<5	1 (12.5)	1 (6.2)	1 (8.3)		1 (7.7)	0 (0.0)	
** **	≥5	7 (87.5)	15 (93.8)	11 (91.7)		12 (92.3)	3 (100.0)	
** **	Missing	5	13	6		12	1	
**Albumin**, No. (%), g/L					0.1336			1.0000
** **	<30	6 (54.6)	20 (69.0)	9 (40.9)		16 (69.6)	4 (66.7)	
** **	≥30	5 (45.4)	9 (31.0)	13 (59.1)		7 (30.4)	2 (33.3)	
** **	Missing	7	14	11		11	3	
**C-reactive protein/albumin ratio**, median [IQR]		1.14 [0.38 – 3.53]	0.73 [0.30 – 1.48]	0.59 [0.24 – 3.83]	0.7645	0.67 [0.27 – 1.43]	1.03 [0.43 – 1.53]	0.7921
	Missing	7	19	11		16	3	
**Follow-up parameters**								
**Median follow-up time** [IQR], months		All patients were followed until death (maximum time observed = 44.7 months) except 2 censored patients with a median follow-up equal to 4.6 months	40.3 [31.7 – 106.5]	60.6 [35.0 – 89.5]		86.7 [10.3 – 106.5]	31.7 [1.12 – 40.25]	

^†^χ^2^ tests or Fisher’s exact tests used to compare proportions, and Wilcoxon tests used to compare continuous variables between the groups according to lymphopenia and sarcopenia.

^‡^χ^2^ tests or Fisher’s exact tests used to compare proportions, and Wilcoxon tests used to compare continuous variables between the groups according to the degree of sarcopenia.

All statistical tests were two-sided.

IQR, Interquartile Range; CA 19-9, Carbohydrate Antigen 19-9; CEA, carcinoembryonic antigen; NA, not available.

**Figure 3:**
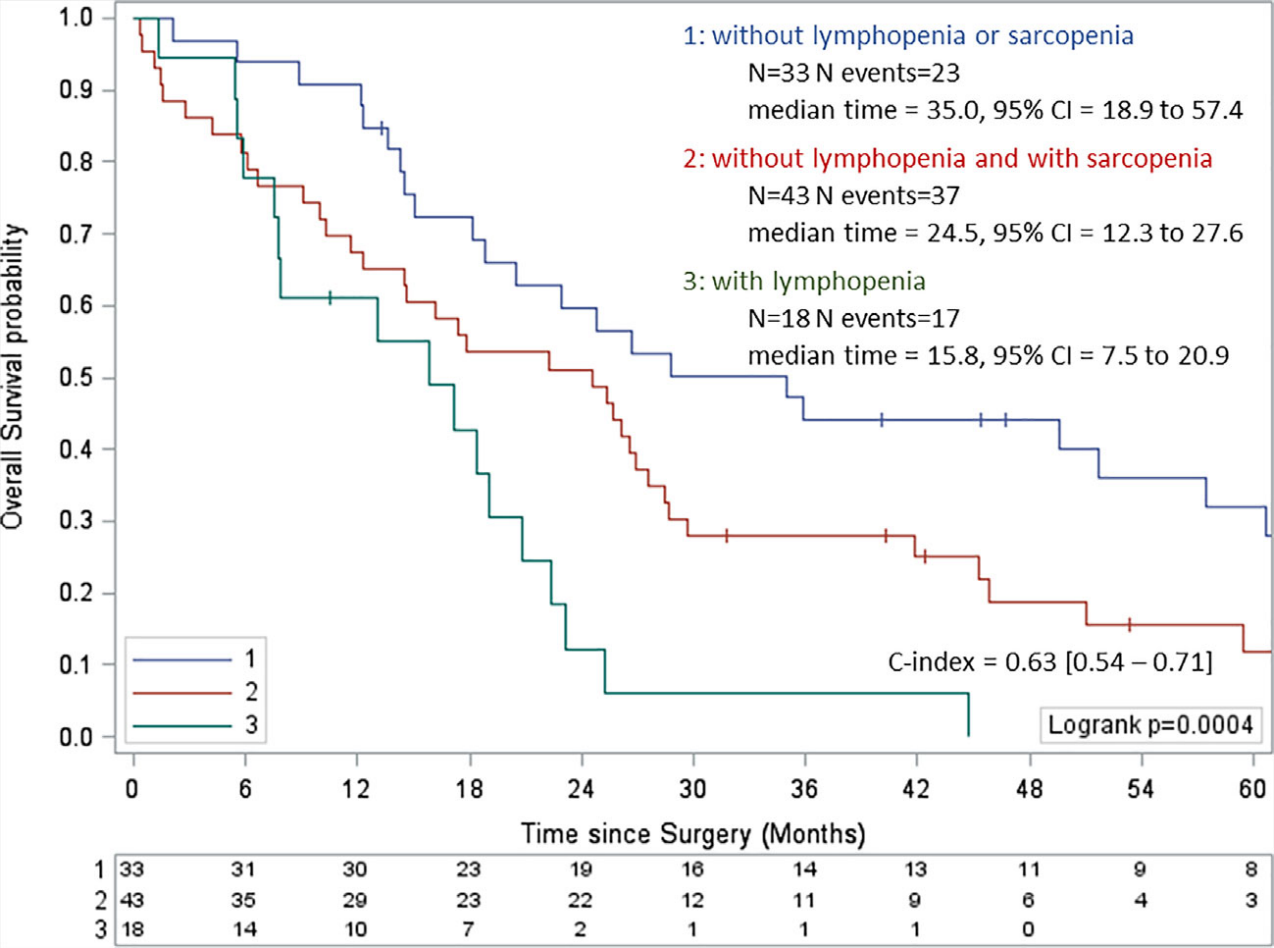
Kaplan-Meier curves of overall survival according to preoperative lymphopenia and sarcopenia. Values of the log-rank test P <0.05 were considered statistically significant, and all tests were two-sided. CI, confidence interval.

The discriminative ability of the three-group model was confirmed in OS analysis (**Figure 3**). Of note, the adjuvant chemotherapy administration was homogeneous in the three risk groups regardless of lymphopenia or sarcopenia levels (p = 0.1557; **Supplementary Table 1**).

### Defining a Threshold of Preoperative Sarcopenia Correlated With PDAC Patients’ Risk of Death

**Figure 4:**
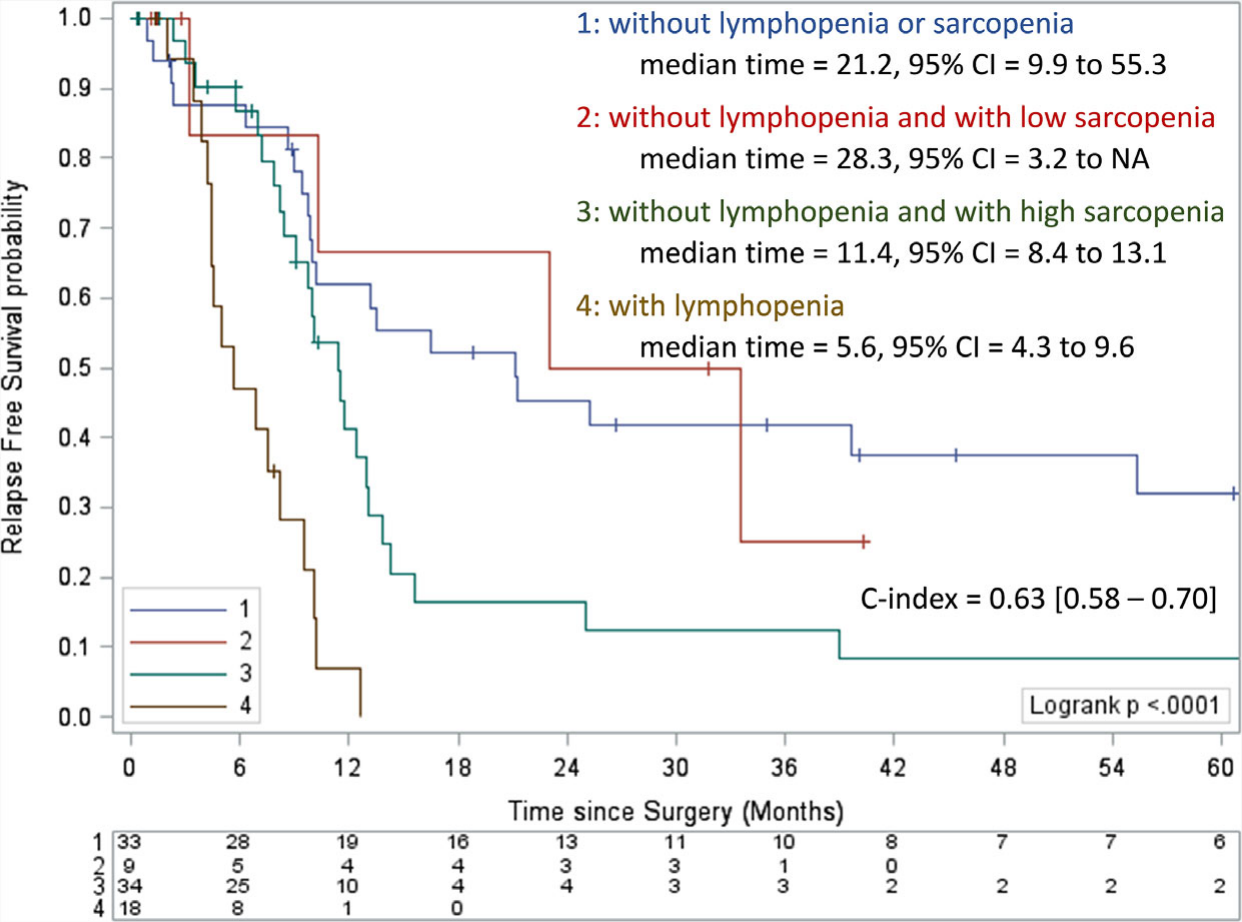
Kaplan-Meier curves of relapse-free survival according to preoperative lymphopenia and the level of sarcopenia. Values of the log-rank test P <0.05 were considered statistically significant, and all tests were two-sided. CI, confidence interval; NA, not available.

In order to determine which threshold of sarcopenia might influence PDAC patients’ clinical outcomes, we explored the prognosis of patients who had no baseline lymphopenia and were clustered in two different groups according to sarcopenia levels. In a first analysis, we observed that the median value for sarcopenia measures could not distinguish different risk groups (**Supplementary Figure 6**). However, using the Cox’s method, two degrees of sarcopenia were associated with prognosis using thresholds of 36.1 cm²/m² for women and 45.7 cm²/m² for men. Thus, different risk groups for RFS were distinguished (**Figure 4**), with a median RFS of 11.4 months (95%CI = 8.4 to 13.1), and 28.3 months (95% CI = 3.2 to NA), respectively (p <0.0001; **Figure 4**). The two risk groups displayed similar patient characteristics (**Table 4**), suggesting that in the absence of lymphopenia, sarcopenia is one of the major determinants to predict the risk of death for patients eligible for PDAC surgery.

### Evaluation of Postoperative Lymphopenia

**Figure 5:**
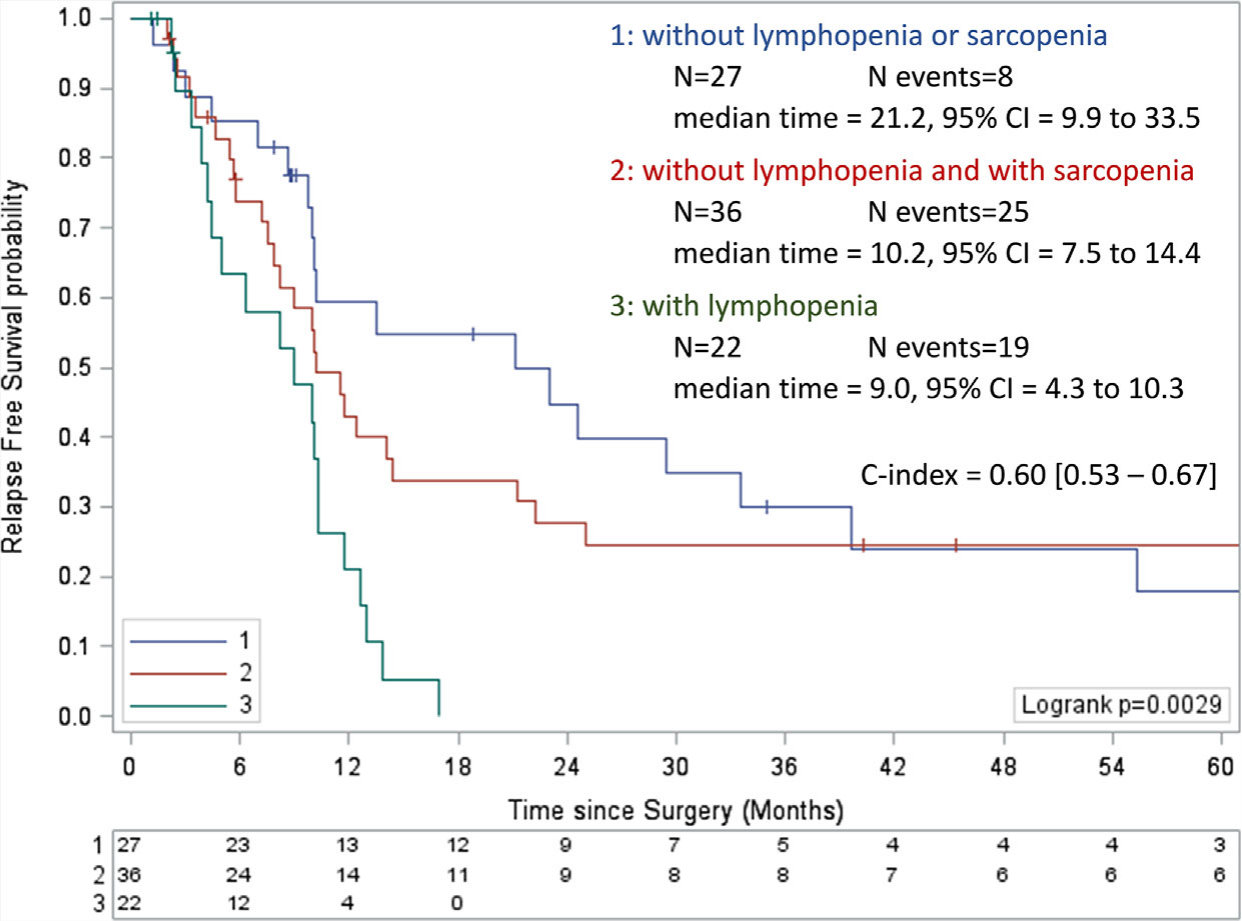
Kaplan-Meier curves of relapse-free survival according to postoperative lymphopenia and sarcopenia. Values of the log-rank test P <0.05 were considered statistically significant, and all tests were two-sided. CI, confidence interval; NA, not available.

We have previously shown that lymphopenia exhibits a better accuracy when monitored one month after rather than before PDAC surgery (10). Then, we decided to analyze the impact of sarcopenia on the incidence of postoperative lymphopenia. First, we confirmed that postoperative lymphopenia was a negative prognostic factor for RFS in the present cohort, in univariate Cox analysis (HR = 2.50, 95%CI = 1.53 to 4.09, p = 0.0003). The preoperative scoring system was applied with postoperative parameters. Similarly, patients were categorized into the same three risk groups (high, intermediate, and low risk) previously identified with statistically significantly different prognostic profiles. This analysis confirmed that patients with lymphopenia had the poorest prognostic, median RFS of 9.0 months (95%CI = 4.3 to 10.3 months; **Figure 5**).

Furthermore, among patients with baseline lymphocyte count ≥ 1,000/mm^3^, the risk of postoperative lymphopenia was significantly enhanced in the presence of sarcopenia measured at diagnosis (77.8% *versus* 22.2%, p = 0.0029; **Table 1**).

## Discussion

Preoperative lymphopenia and sarcopenia were identified as independent prognostic factors for RFS in LPDAC. The additive value of baseline sarcopenia and lymphopenia allows the proposal of a prognostic score where LPADC patients are classified into three risk groups. These results highlight considerable heterogeneity in LPDAC patients’ survival.

Our study confirmed that preoperative lymphocyte count is an independent prognostic factor in LPDAC (HR = 4.57, p <0.0001). The use of a threshold offered better discrimination than the use of lymphocyte count because it allows relapse-risk stratification. Both lymphocyte count and NLR are recognized independent prognostic factors in pancreatic adenocarcinoma and are used in clinical practice (24, 25).

In our cohort, the prevalence of sarcopenia was high (60%), as observed in previous studies (12, 26). Median BMI was 24.8 kg/m² (IQR, 22.2 – 27.7) and more than half of patients (52.1%) were overweight or obese. Importantly, almost 40% of these individuals had also preoperative sarcopenia. Severe depletion of skeletal muscle detection remains a challenge in patients with obesity (27). BMI and clinical evaluation are not enough, a radiological assessment of skeletal muscle area is necessary to identify sarcopenic obesity. We highlighted that preoperative sarcopenia is the only nutritional independent prognostic factor for RFS in LPDAC (HR = 1.78, p = 0.0469). Few studies have analyzed the prognostic value of preoperative sarcopenia in LPDAC on OS and RFS and showed contradictive results (26, 28–30), probably because of the lack of consensus on the definition of sarcopenia. Among many definitions, we chose to use the threshold defined by Prado et al. (31), validated for gastrointestinal cancer and in the western population, as ours. Nevertheless, we pointed out a new cut off value of sarcopenia more accurately correlated to prognosis with thresholds corresponding to 36.1 cm²/m² for women and 45.7 cm²/m² for men to distinguish high-risk group with a median RFS of 11.4 months and low-risk group with a median RFS of 28.3 months (P <0.0001).

Prognostic factors that can be identified before surgery and chemotherapy are mandatory to stratify the treatment decision-making process in current clinical practice and for the development of more personalized neoadjuvant strategies. In the multivariate analysis performed in the present study, only preoperative lymphocyte count and sarcopenia were independent prognostic factors. Thus, combining both variables allowed to elaborate a preoperative prognostic score and identified several subgroups of patients with different prognoses. Patients with the worse prognosis were those with lymphopenia (RFS of 5.6 months, 95%CI = 4.3 to 9.6 months).

Similarly, postoperative lymphopenia is also an independent negative prognostic factor (HR = 2.50, 95%CI = 1.53 to 4.09, p = 0.0003). In postoperative time, the score confirmed that patients with lymphopenia had the worst clinical outcomes, with a median RFS of 9.0 months (95%CI = 4.3 to 10.3 months). In our previous study, none of postoperative lymphopenic patients had long-term survival (10). Similarly, in the study of Tsujita et al. (32), the 3 years survival rate after pancreatectomy was 33.9% in patients with a postoperative NLR of less than 3 at one month and 7.3% in those with a postoperative NLR of 3 or more (p <0.001). Interestingly, in our study, 77.8% of patients with postoperative lymphopenia had preoperative sarcopenia suggesting the predictive value of this factor. Our results suggest that sarcopenia measured at the baseline might be a predictive factor for the occurrence of post-operative lymphopenia.

Several mechanisms are probably involved in sarcopenia (a decreased of skeletal muscle mass). Inadequate intake due to anorexia, increased energy expenditure, systemic inflammation, and abnormal metabolism result in muscle wasting and body weight loss (33). In addition, tumor cells product pro-cachectic factors such as proteolysis-inducing factor (34) and also interact with host cells to produce inflammatory cytokines, such as TNF-α, IL-1, and IL-6 which activate muscular nuclear factor-kappa β (NF-κβ) and cause wasting of skeletal muscle (35, 36) notably in pancreatic cancer (37). Some measures that have been proposed to treat sarcopenia have not been supported by evidence and currently, no study has shown an increase in lean mass nor OS following the usual nutritional treatments in pancreatic cancer (38). However, according to Sandini et al., after neoadjuvant chemotherapy some patients with primary unresectable pancreatic cancer who underwent resection had experienced a 5.9% skeletal muscle area increase during treatment, whereas those who did not undergo resection had a 1.7% decrease (p <0 .001) (39).

Decreased lymphocyte count results from an inadequate immunologic reaction and is a valuable biomarker for identifying cancers associated with an increased risk of tumor immune evasion and poor prognosis. The role of the immune system in cancer was highlight by studies investigating the prognostic influence of Tumor Infiltrating Lymphocytes (TIL). Indeed, in pancreatic cancer elevated CD8^+^ T lymphocytes in tumor stroma is a favorable prognostic factor influencing OS. Conversely, an increasing rate of FOXP3^+^ lymphocytes reflects immunological tolerance and correlates with decreased survival rates (40, 41). Interstingly accumulating data in immunology attested that chemotherapy might improve anti-tumor immunity (42). In breast cancer, Goto et al. point out the predictive value of change in the CD8^+^ TIL levels and the CD8/FOXP3 ratio (p <0.001) after neoadjuvant therapy (43). In pancreatic cancer, after neoadjuvant chemotherapy, the median OS of patients with a high CD8^+^/FOXP3^+^ lymphocyte ratio was longer than that of patients with a low CD8^+^/FOXP3^+^ lymphocyte ratio (p=0.01) (44).

These data suggest the potential utility of neoadjuvant strategy in LPDAC patients with preoperative lymphopenia and/or sarcopenia. Some prehabilitation studies including nutrition and exercise are in progress and may impact sarcopenia, lymphopenia, and probably quality of life (45). In addition, the available data suggest a potential anti-tumor effect of the practice of physical activity and a benefit on survival, which could be mediated in particular by the decrease in insulin resistance, the modulation of the secretion of adiponectins, the decrease of the inflammatory syndrome, a modulating effect of intratumoral signaling pathways, a decrease in the toxicity of the treatments and therefore a better dose-intensity, and the reduction of sarcopenia (46–48). Systemic inflammation can be also reduced by pharmacological agents (such as corticosteroids or nonsteroidal anti-inflammatory drugs) as well as specific nutrients enriched with fatty acids. Particularly, some protocols with omega-3 fatty acids are under investigation in elderly patients (49).

There are some limitations in our study. There are some missing data due to the retrospective design of the study, but the two groups with or without complete information displayed similar RFS (**Supplementary Figure 3**). Our results have to be confirmed using a validation cohort. From a statistical point of view, the assessment of model performance measures such as discrimination, calibration, and internal validation strengthen the present investigation. The multivariate analysis significantly improved the model discrimination capacity because the C statistic increased significantly from 0.60 to 0.67 (bootstrap mean difference = 0.07, 95% CI = 0.57 to 0.77) demonstrating the additive value of lymphopenia and sarcopenia for death risk stratification. Moreover, the assessment of skeletal muscle area is only quantitative. The quality of the muscle (skeletal muscle density) and muscle function (handgrip strength are not evaluated, but these measurements are strongly correlated with muscle mass and associated with survival in digestive cancers (50, 51).

Finally, our results may provide evidence for appropriate lymphocyte count and sarcopenia cut-off definition in order to better select PDAC patients eligible for neoadjuvant therapy. Preoperative lymphopenia and sarcopenia are pejorative independent prognostic factors for RFS and OS in LPDAC. Assessment of these factors at baseline may be relevant in current clinical practice for death risk stratification.

## Data Availability Statement

The raw data supporting the conclusions of this article will be made available by the authors, without undue reservation.

## Ethics Statement

The studies involving human participants were reviewed and approved by National French Commission for bioinformatics data and patient liberty (CNIL). The patients/participants provided their written informed consent to participate in this study.

## Author Contributions

Conception and design: Cd’E, JG, JR, CB, and AV. Administrative support: DV, CB, and AV. Provision of study materials or patients: Cd’E, JG, JR, BH, CB, and AV. Collection and assembly of data: Cd’E, JG, JR, and AV. Data analysis and interpretation: Cd’E, JG, JR, DV, CB, and AV. Manuscript writing: All authors. All authors contributed to the article and approved the submitted version.

## Conflict of Interest

The authors declare that the research was conducted in the absence of any commercial or financial relationships that could be construed as a potential conflict of interest.

## Abbreviations

95%CI, 95% confidence intervals; CA19-9, carbohydrate antigen 19-9; CEA, carcinoembryonic antigen; CNIL, National French Commission for bioinformatics; CT, computed tomography; HR, hazard ratio; IL-1, interleukin 1; IL-6, interleukin 6; IMA, index muscular area; IQR, interquartile range; L3, third lumbar vertebral level; LPDAC, localized pancreatic ductal adenocarcinoma; NA, not available; NF-κβ, nuclear factor-kappa β; NLR, neutrophil to lymphocyte ratio; OS, overall survival; PDAC, pancreatic ductal adenocarcinoma; RFS, relapse-free survival; SM, skeletal muscle mass; TNF-α, tumor necrosis factor alpha.
