# Supplementary material for: Additive Value of Preoperative Sarcopenia and Lymphopenia for Prognosis Prediction in Localized Pancreatic Ductal Adenocarcinoma
Source: Front Oncol. 2021 May 27;11:683289. doi: 10.3389/fonc.2021.683289 (PMC8190386; doi:10.3389/fonc.2021.683289)
Supplement: Supplementary file 1 [file DataSheet_1.pdf]

SUPPLEMENTARY MATERIAL

Additive value of preoperative sarcopenia and lymphopenia for prognosis prediction in localized pancreatic ductal adenocarcinoma

Running title: Preoperative sarcopenia and lymphopenia in pancreatic cancer

Christelle d'Engremont[1†], Julienne Grillot[1†], Julie Raillat[1], Dewi Vernerey[2], Lucine Vuitton[1], Stéphane Koch[1], Célia Turco[3], Bruno Heyd[3], Guillaume Mouillet[4], Quentin Jacquinot[4], Christophe Borg[4-6], Angélique Vienot[4-6]

[†] These authors have contributed equally to this work and share first authorship

Affiliations:

1. Department of Gastroenterology and Nutrition, University Hospital of Besançon, F-25000 Besançon, France
2. Methodology and Quality of Life in Oncology Unit, University Hospital of Besançon, F-25000 Besançon, France
3. Department of Digestive Surgery and Liver Transplantation, University Hospital of Besançon, F-25000 Besançon, France
4. Department of Medical Oncology, University Hospital of Besançon, F-25000 Besançon, France
5. INSERM, EFS BFC, UMR1098, RIGHT, University of Bourgogne Franche-Comté, Interactions Greffon-Hôte-Tumeur/Ingénierie Cellulaire et Génique, F-25000 Besançon, France
6. Clinical Investigational Center, CIC-1431, F-25000 Besançon, France

Corresponding author:

Angélique Vienot, MD, Department of Medical Oncology, University Hospital of Besançon, F-25000 Besançon, France;

Tel.: +33 370 632 278, Fax: +33 370 632 214,

E-mail: a3vienot@chu-besancon.fr

<u>Supplementary Methods</u>: Statistical analyses interpretation

Discrimination

The discrimination refers to the ability of separating patients with different prognosis. The C-index estimates the proportion of all pairwise patient combinations from the sample data whose survival time can be ordered according to whether the patient with the highest predicted survival is the one who actually survived longer (discrimination). The C-index ($0 \leq C \leq 1$) is a probability of concordance between predicted and observed survival, with C-index = 0.5 for random predictions and C-index = 1 for a perfectly discriminating model. In this study, the C-index calculation was repeated 1,000 times in random samples of the initial dataset with the use of bootstrap sampling procedures to derive 95% percentile confidence intervals for the C-index.

Calibration

Calibration and goodness-of-fit refer to the ability to provide unbiased survival predictions in groups of similar patients. A prediction model is considered “well-calibrated” if the difference between predictions and observations in all groups of similar patients is close to 0 (perfect calibration).

Bootstrapping

Bootstrapping is the preferred simulation technique that was first described by Bradley Efron (1). The original dataset is a random sample of patients being representative of a general population. Bootstrapping means generating a large number of datasets, each of which with the same sample size as the original one, by resampling with replacement (i.e., a previously selected patient may be selected again).

Internal validation

Internal validation is useful to obtain an honest estimation of the model performance for patients that are similar to those in the development sample and to indicate an upper limit to the expected performance in other settings. The bootstrap approach is the preferred technique to assess internal validity.

References

1. Efron B. Bootstrap Methods: Another Look at the Jackknife. Ann Stat 19797 1–26.

Supplementary Figures

Supplementary Figure 1. Restricted cubic spline modelization.

Abbreviations: CA 19-9=Carbohydrate Antigen 19-9

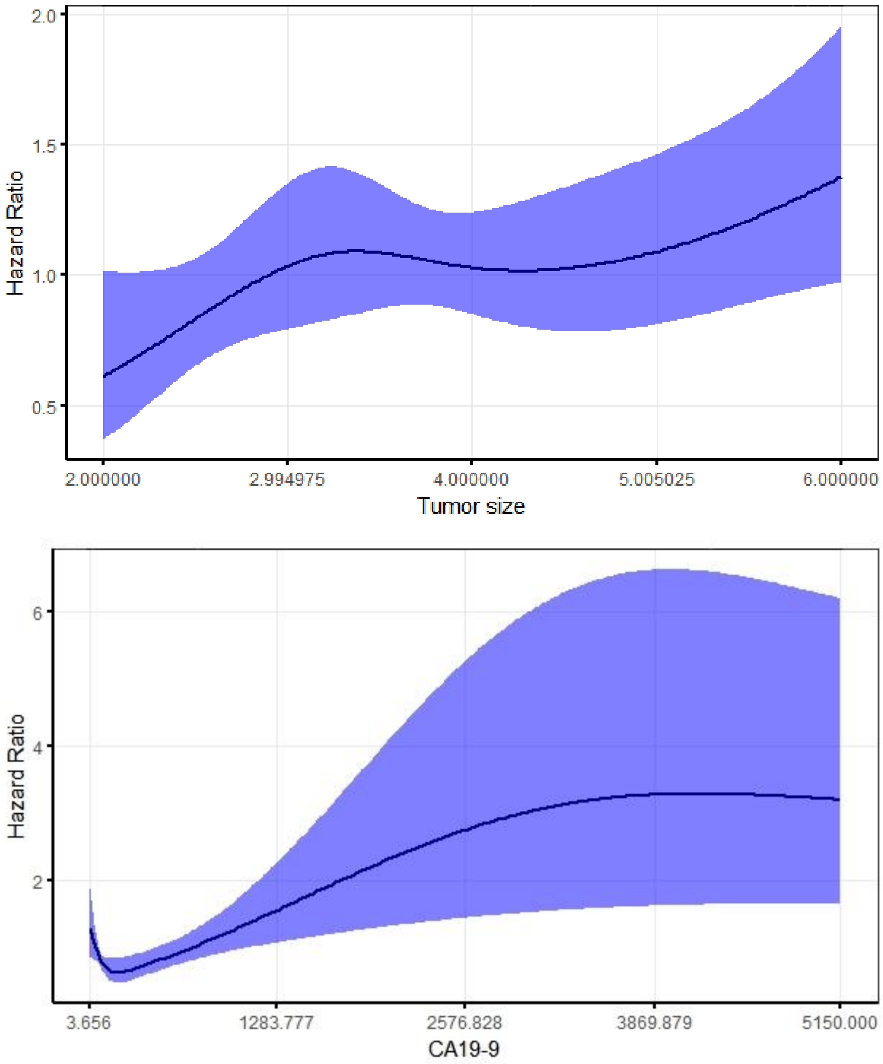

Supplementary Figure 2. Correlation matrix.

Pearson correlation coefficients: < 0.2 – 0.2-0.4 – ≥ 0.4 P-value: > 0.001 – ≤ 0.001

Abbreviations: CA 19-9=Carbohydrate Antigen 19-9

	Tumor size	Skeletal muscle mass index	Sarcopenia	Lymphopenia	Neutrophil-to-lymphocyte ratio	CA19-9
**Tumor size**	1.00000	-0.08986	0.06430	0.05328	0.08706	0.32000
		0.4024	0.5494	0.5519	0.3363	0.0007
	134	89	89	127	124	108
**Skeletal muscle mass index**	-0.08986	1.00000	-0.53480	0.06018	-0.04830	-0.12346
	0.4024		<.0001	0.5645	0.6475	0.2632
	89	98	98	94	92	84
**Sarcopenia**	0.06430	-0.53480	1.00000	-0.08056	0.13014	0.04974
	0.5494	<.0001		0.4402	0.2163	0.6532
	89	98	98	94	92	84
**Lymphopenia**	0.05328	0.06018	-0.08056	1.00000	-0.58428	-0.08205
	0.5519	0.5645	0.4402		<.0001	0.3920
	127	94	94	139	135	111
**Neutrophil-to-lymphocyte ratio**	0.08706	-0.04830	0.13014	-0.58428	1.00000	0.27654
	0.3363	0.6475	0.2163	<.0001		0.0038
	124	92	92	135	135	108
**CA19-9**	0.32000	-0.12346	0.04974	-0.08205	0.27654	1.00000
	0.0007	0.2632	0.6532	0.3920	0.0038	
	108	84	84	111	108	118

Supplementary Figure 3. Kaplan-Meier curves of relapse-free survival according to the groups with or without missing data regarding to preoperative lymphopenia and sarcopenia.

Values of the log-rank test P <0.05 were considered statistically significant, and all tests were two-sided.

Abbreviations: CI = confidence interval.

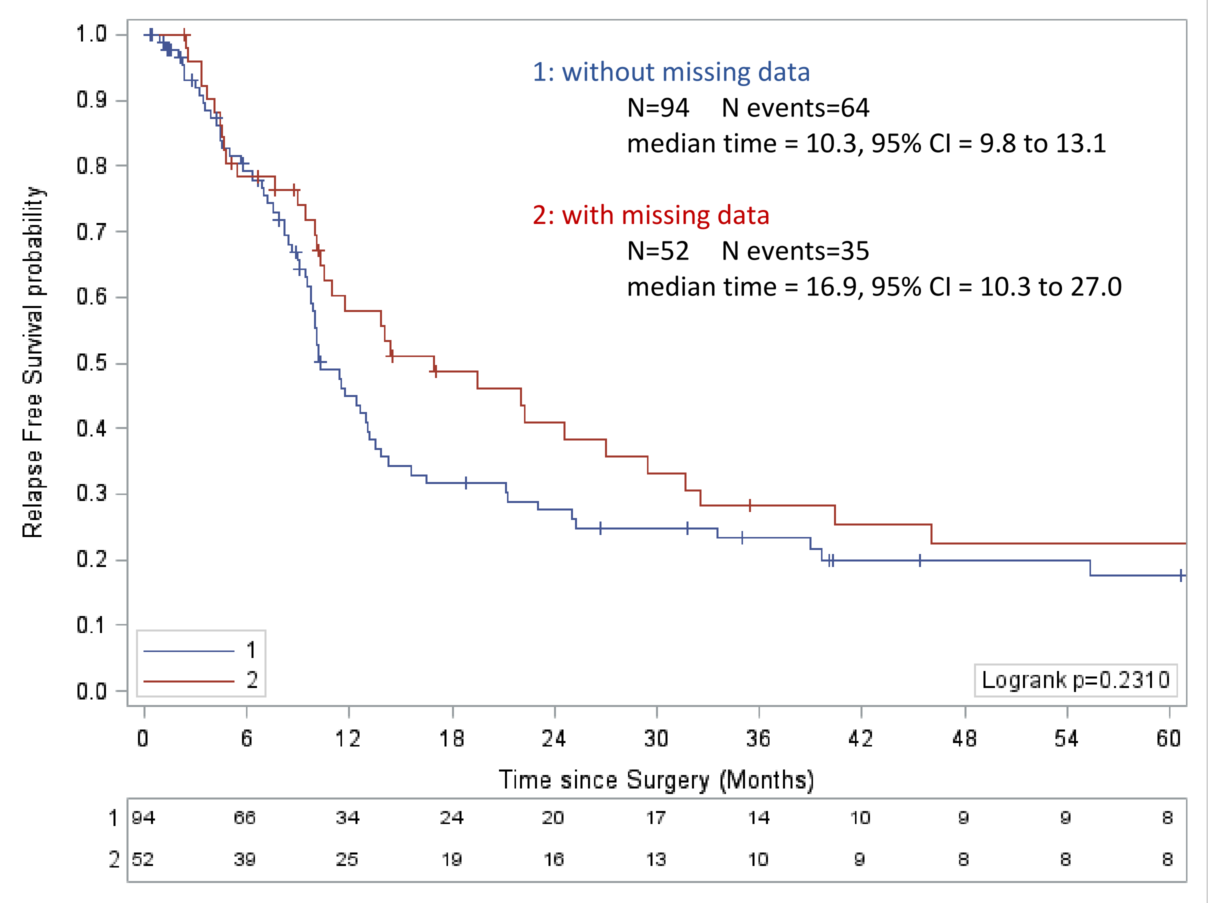

Supplementary Figure 4. Calibration plots at 6, 12, 24, and 48 months for the final multivariate model.

Vertical axis is the observed proportion of patients surviving at time of interest. Black line=observed; Grey line=ideal calibrated model; Blue line= bootstrap corrected estimates (optimism corrected). B= 20 repetitions for bootstrap.

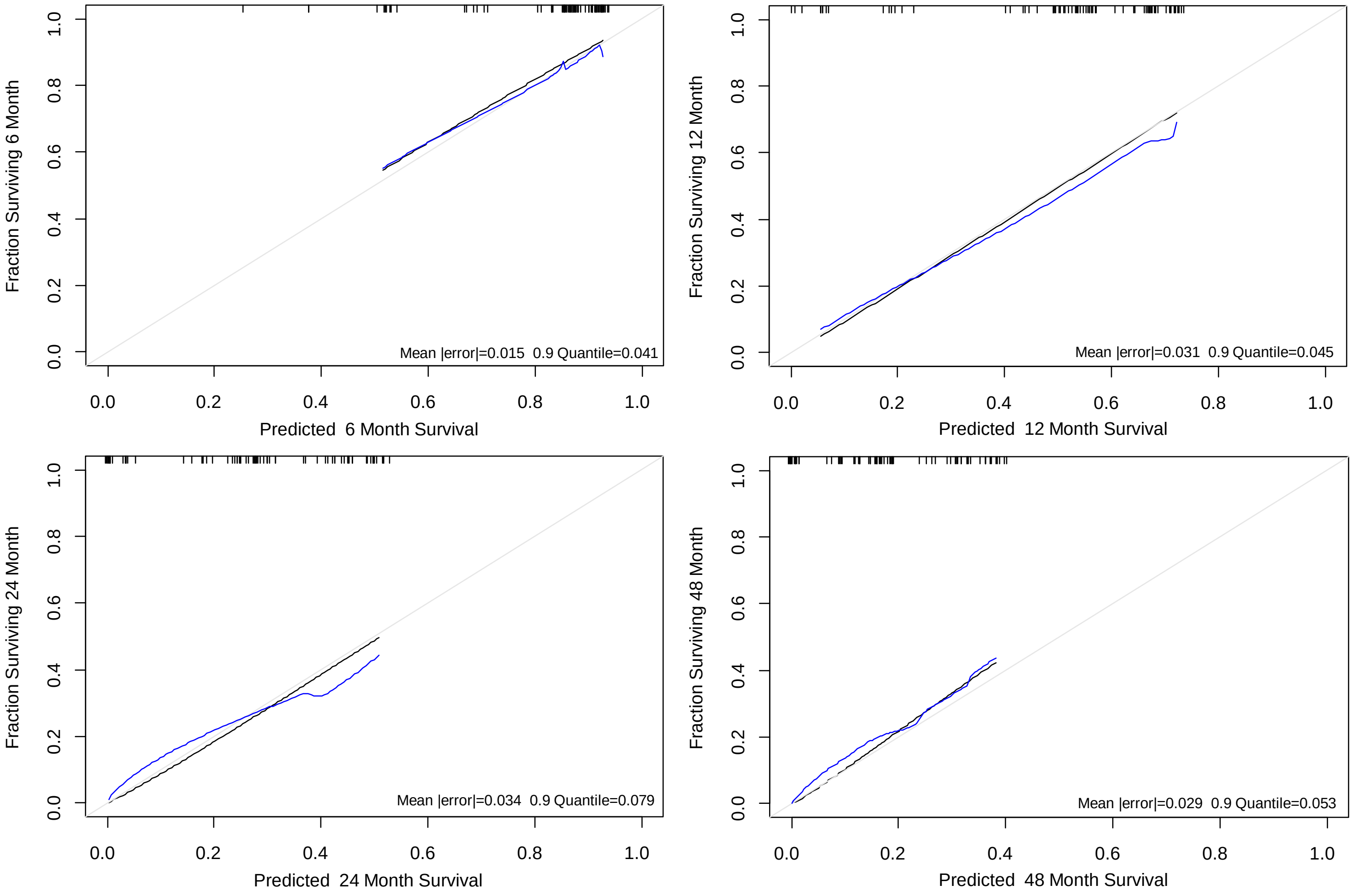

Supplementary Figure 5. Kaplan-Meier curves of relapse-free survival according to preoperative lymphopenia and sarcopenia with four groups.

Values of the log-rank test P <0.05 were considered statistically significant, and all tests were two-sided.

Abbreviations: CI = confidence interval.

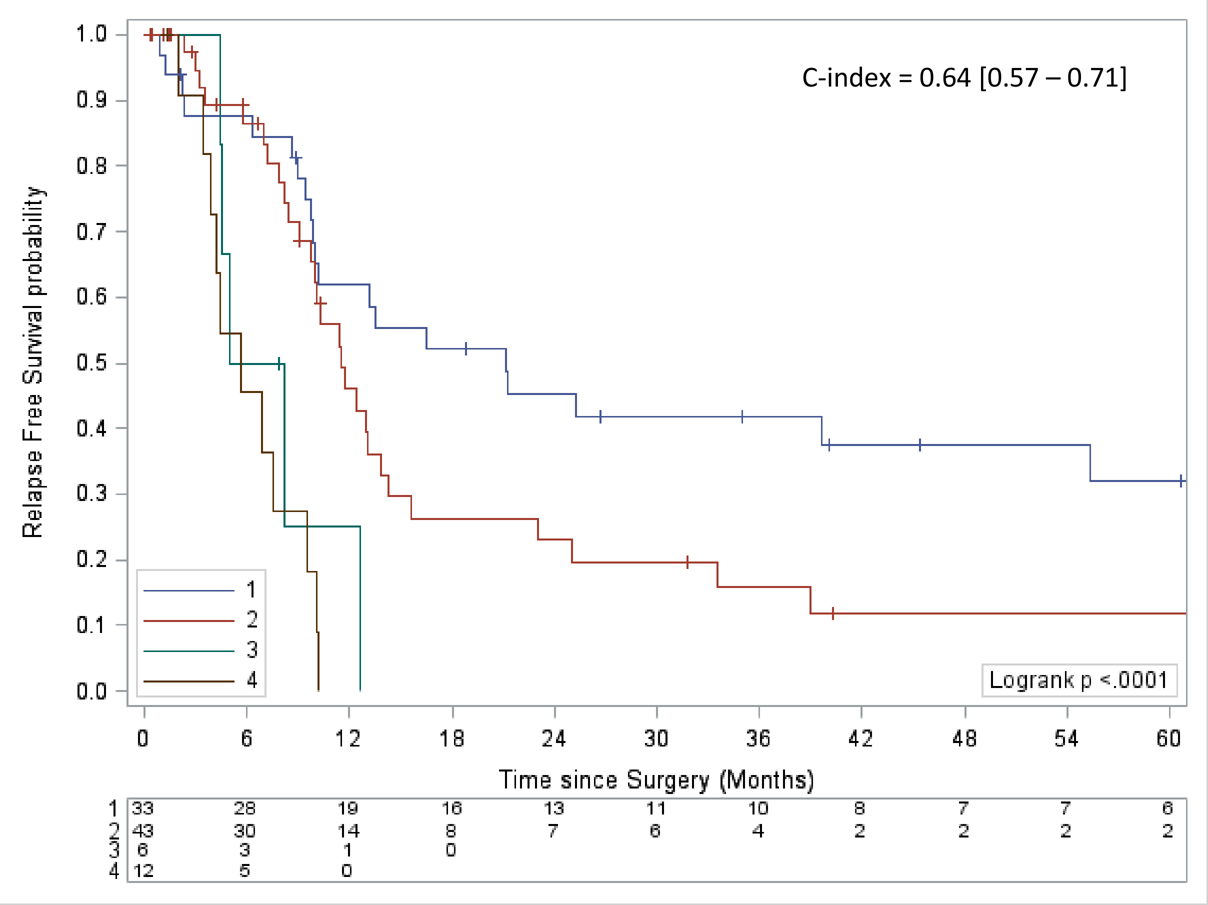

1: without lymphopenia or sarcopenia

N=33 N events=20

median time = 21.2, 95% CI = 9.9 to 55.3

2: without lymphopenia and with sarcopenia

N=43 N events=28

median time = 11.5, 95% CI = 9.8 to 13.9

3: with lymphopenia and without sarcopenia

N=12 N events=11

median time = 5.6, 95% CI = 3.4 to 9.6

4: with lymphopenia and with sarcopenia

N=6 N events=5

median time = 6.6, 95% CI = 4.4 to 12.6

Supplementary Figure 6. Kaplan-Meier curves of relapse-free according to preoperative lymphopenia and the level of sarcopenia defined by median.

Values of the log-rank test P <0.05 were considered statistically significant, and all tests were two-sided.

Abbreviations: CI = confidence interval.

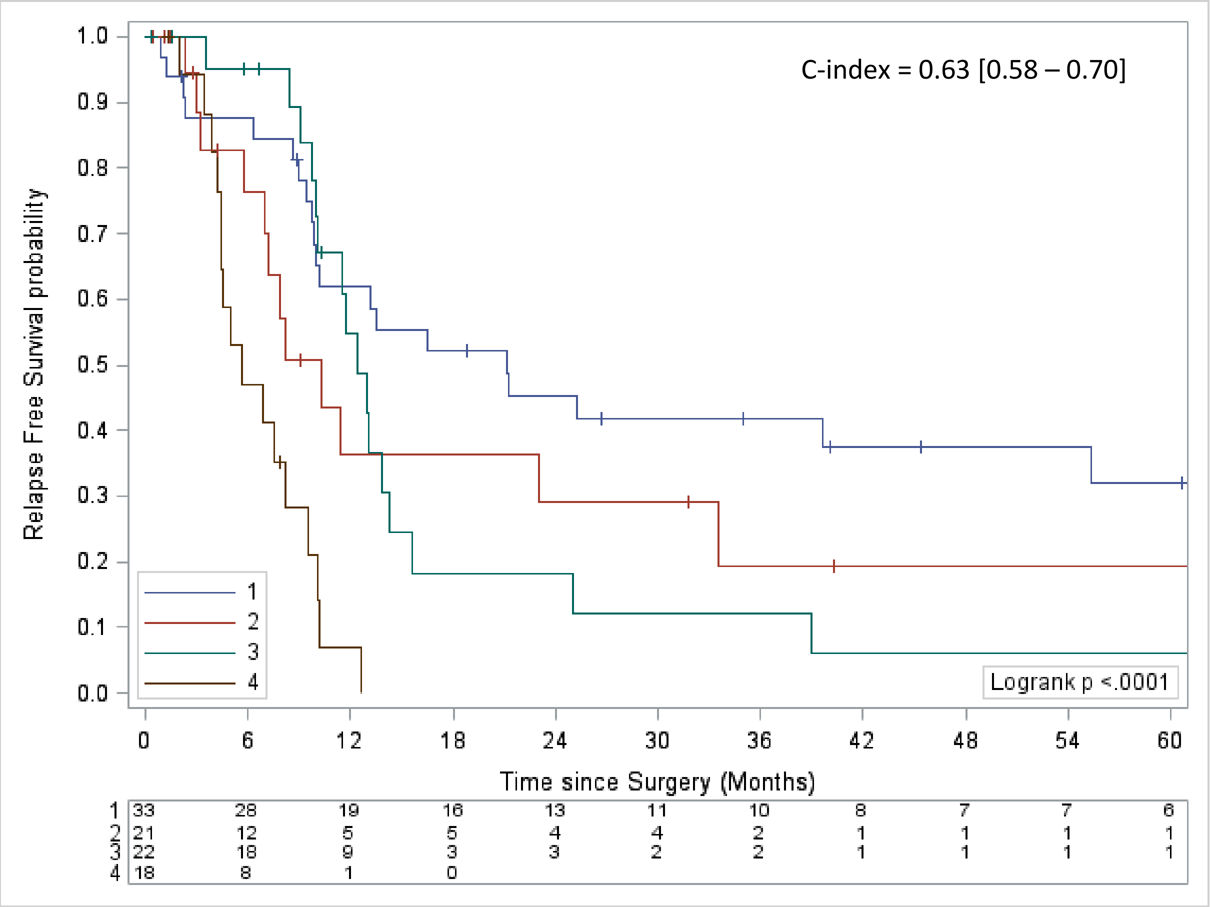

1: without lymphopenia or sarcopenia

N=33 N events=20

median time = 21.2, 95% CI = 9.9 to 55.3

2: without lymphopenia and with low sarcopenia

N=21 N events=12

median time = 10.3, 95% CI = 5.8 to 33.5

3: without lymphopenia and with high sarcopenia

N=22 N events=16

median time = 12.4, 95% CI = 10.0 to 14.3

4: with lymphopenia

N=18 N events=16

median time = 5.6, 95% CI = 4.3 to 9.6

Supplementary Tables

Supplementary Table 1. Adjuvant chemotherapy characteristics in each risk group

Characteristics	Patients with lymphopenia (n = 18)	Patients without lymphopenia		P†	Patients without lymphopenia		P‡
		With sarcopenia (N = 43)	Without sarcopenia (N = 33)		With high sarcopenia (N = 34)	With low sarcopenia (N = 9)	
**Adjuvant chemotherapy**							
**Time between surgery and chemotherapy**, median [IQR], days	57.0 [48.0 – 75.0]	62.0 [54.0 – 76.0]	62.5 [50.5 – 78.8]	0.8545	62.0 [54.0 – 76.0]	65.5 [51.5 – 74.5]	1.0000
Missing	3	14	5		9	5	
**Administration**, No. (%)				0.1557			0.1242
No	3 (16.7)	14 (32.6)	5 (15.1)		9 (26.5)	5 (55.6)	
Yes	15 (83.3)	29 (67.4)	28 (84.9)		25 (73.5)	4 (44.4)	
**Type of chemotherapy**, No. (%)				1.0000			1.0000
Gemcitabine	15 (100.0)	27 (93.1)	28 (100.0)		23 (92.0)	4 (100.0)	
Other	0 (0.0)	2 (6.9)	0 (0.0)		2 (8.0)	0 (0.0)	
- *LV5FU2*||	*0 (0.0)*	*0 (0.0)*	*0 (0.0)*		*0 (0.0)*	*0 (0.0)*	
- *GEMOX¶*	*0 (0.0)*	*1 (3.5)*	*0 (0.0)*		*1 (4.0)*	*0 (0.0)*	
- *CAPOX#*	*0 (0.0)*	*0 (0.0)*	*0 (0.0)*		*0 (0.0)*	*0 (0.0)*	
- *GEM-CAP***	*0 (0.0)*	*1 (3.5)*	*0 (0.0)*		*1 (4.0)*	*0 (0.0)*	
Missing	3	14	5				
**Duration of chemotherapy**, median [IQR], months	2.8 [2.1 – 5.1]	5.1 [3.9 – 5.5]	5.1 [3.4 – 5.3]	0.2820	5.1 [3.9 – 5.5]	4.4 [2.5 – 5.3]	0.5116
Missing	3	14	5		9	5	
**Number of cures**, median [IQR]	10.0 [8.0 – 18.0]	17.0 [14.0 – 18.0]	18.0 [11.5 – 18.0]	0.5241	17.0 [14.0 – 18.0]	16.0 [9.5 – 18.0]	0.7927
Missing	3	14	5		9	5	
**Discontinuation of chemotherapy**, No. (%)				0.5355			1.0000
No	7 (46.7)	17 (58.6)	18 (64.3)		15 (60.0)	2 (50.0)	
Yes	8 (53.3)	12 (41.4)	10 (35.7)		10 (40.0)	2 (50.0)	
Missing	3	14	5				
**Reason for discontinuation**, No. (%)				0.1934			0.4697
Progression disease	4 (50.0)	3 (25.0)	1 (12.5)		2 (20.0)	1 (50.0)	
Toxicity	4 (50.0)	4 (33.3)	5 (62.5)		3 (30.0)	1 (50.0)	

Other	0 (0.0)	5 (41.7)	2 (25.0)		5 (50.0)	0 (0.0)	
Missing	10	31	25		24	7	
**Toxicity (grade ≥2)**, No. (%)				0.9915			1.0000
No	10 (76.9)	25 (75.8)	18 (75.0)		20 (74.1)	5 (83.3)	
Yes	3 (23.1)	8 (24.2)	6 (25.0)		7 (25.9)	1 (16.7)	
- *Hematology*	*2 (15.4)*	*5 (15.1)*	*2 (8.3)*		*4 (14.8)*	*1 (16.7)*	
- *Digestive*	*0 (0.0)*	*0 (0.0)*	*1 (4.2)*		*0 (0.0)*	*0 (0.0)*	
- *Other*	*1 (7.7)*	*3 (9.1)*	*3 (12.5)*		*3 (11.1)*	*0 (0.0)*	
Missing	5	10	9		7	3	

† χ^2 tests or Fisher's exact tests used to compare proportions, and Wilcoxon tests used to compare continuous variables between the groups according to lymphopenia and sarcopenia.

‡ χ^2 tests or Fisher's exact tests used to compare proportions, and Wilcoxon tests used to compare continuous variables between the groups according to the degree of sarcopenia.

All statistical tests were two-sided.

Abbreviations: IQR=Interquartile Range

|| LV5FU2=5-fluorouracil [5-FU], levofolinate

¶ GEMOX=gemcitabine, oxaliplatine

CAPOX=capecitabine, oxaliplatine

** GEM-CAP=gemcitabine, capecitabine
